# Sensitivity and Specificity of Human Papillomavirus (HPV) 16 Early Antigen Serology for HPV-Driven Oropharyngeal Cancer: A Systematic Literature Review and Meta-Analysis

**DOI:** 10.3390/cancers13123010

**Published:** 2021-06-16

**Authors:** Julia Hibbert, Gordana Halec, Dan Baaken, Tim Waterboer, Nicole Brenner

**Affiliations:** 1 Infections and Cancer Epidemiology Division, German Cancer Research Center (DKFZ), 69120 Heidelberg, Germany; g.halec@dkfz-heidelberg.de (G.H.); t.waterboer@dkfz.de (T.W.); 2 Institute of Medical Biostatistics, Epidemiology and Informatics (IMBEI), University Medical Center of the Johannes Gutenberg-University, 55131 Mainz, Germany; dabaaken@uni-mainz.de

**Keywords:** HPV, serology, antibodies, sensitivity, specificity, oropharyngeal cancer, systematic review, meta-analysis

## Abstract

**Simple Summary:**

Serum antibodies against human papillomavirus 16 (HPV16) proteins are associated with HPV-driven oropharyngeal cancer (HPV-OPC). The HPV status of OPC cases is clinically relevant because patients with HPV-OPC show improved survival and treatment response compared to tobacco- or alcohol-induced OPC. In clinical settings, molecular HPV tumor status is usually determined by tissue-based methods detecting molecular markers, such as viral nucleic acids or p16 overexpression. Antibodies against HPV16 in peripheral blood were shown to be very accurate in determining the molecular HPV tumor status in multiple studies. In this work, we reviewed and summarized the available literature on the performance of HPV16 serology for E2, E6 and E7 antibodies to determine molecular HPV tumor status in OPC cases in comparison with tissue-based reference methods. We calculated summary estimates across different studies for sensitivity and specificity, and we investigated factors influencing test performance.

**Abstract:**

Antibodies against HPV16 early proteins have been shown to be promising biomarkers for the identification of HPV-driven oropharyngeal cancer (HPV-OPC) among OPC cases in multiple studies. A systematic literature search was performed to identify original research articles comparing HPV early antigen serology with established reference methods to determine molecular HPV tumor status. Random-effects models were used to calculate summary estimates for sensitivity and specificity of HPV16 E2, E6 and E7 serology for HPV-OPC. Subgroup analyses were performed to explore heterogeneity across studies and describe variables associated with test performance. We identified *n* = 23 studies meeting all eligibility criteria and included these in the meta-analysis. E6 serology showed the best performance with pooled sensitivity and specificity estimates of 83.1% (95% confidence interval (CI) 72.5–90.2%) and 94.6% (95% CI 89.0–97.4%), respectively, while E2 and E7 serological assays were highly specific (E2: 92.5% (95% CI 79.1–97.6%); E7: 88.5% (95% CI 77.9–94.4%)) but moderately sensitive (E2: 67.8% (95% CI 58.9–75.6%); E7: 67.0% (95% CI 63.2–70.6%)). Subgroup analyses revealed increased pooled sensitivity for bacterially (89.9% (95% CI 84.5–93.6%)) vs. in vitro expressed E6 antigen (55.3% (95% CI 41.0–68.7%)), while both showed high specificity (95.2% (95% CI 93.0–96.7%) and 91.1% (95% CI 46.6–99.2%), respectively). Pooled specificity estimates for HPV16 E2, E6 and E7 serology were significantly lower in studies utilizing HPV DNA PCR as the only molecular reference method compared to those using a combination of any two reference methods (HPV DNA, RNA, in situ hybridization (ISH), p16 immunohistochemistry (IHC)), or histopathological reference methods (ISH or p16 IHC) as stand-alone marker. In conclusion, HPV16 E6 seropositivity is a highly sensitive and specific biomarker for HPV-OPC. However, its performance differs between serological assays and depends on molecular reference methods.

## 1. Introduction

The incidence of oropharyngeal cancer (OPC) has been rising over the last decades in many Western countries due to an increase in incident OPC cases attributable to infection with oncogenic human papillomaviruses (HPV) [1]. The HPV attributable fraction (AF) for OPC differs by geographic region and human development index, and ranges between 10% [2] and more than 70% for example, in the US [3]. Among HPV-driven OPC (HPV-OPC), 80–90% are attributable to HPV type 16 (HPV16) alone [4].

HPV-OPC on the one hand, and tobacco- and/or alcohol-induced HPV-negative OPC on the other are considered distinct disease entities as they differ in their epidemiology, molecular characteristics and clinical behavior [5]. HPV status is of prognostic importance in OPC cases because HPV-OPC generally shows a better treatment response [6] and increased overall survival [7] compared to HPV-negative OPC. This led to multiple clinical trials investigating treatment de-escalation for HPV-OPC [8].

Several methods have been established to determine molecular HPV tumor status, each conferring their own advantages and disadvantages. The detection of HPV DNA by polymerase chain reaction (PCR) is considered the least reliable because it only suggests the presence of viral genetic material but does not identify HPV-driven carcinogenic processes marked by active transcription of the HPV oncogenes E6 and E7 [9]. The most common marker in clinical practice, p16 immunohistochemistry (IHC), detects p16^ink4^ overexpression in cancerous cells caused by the E7 oncoprotein [10,11]. However, transforming HPV infections are not the only possible cause of p16^ink4^ overexpression in cancer cells limiting the specificity of this method [10,12]. In situ hybridization (ISH) not only allows the detection but also the localization of the viral genome within a cell, providing more reliable results than mere detection of HPV DNA by PCR, albeit with lower sensitivity [13]. Although all prior mentioned methods show weaknesses when used on their own, their application in combination, such as HPV DNA PCR and p16 IHC, has shown improved performance by increasing specificity while maintaining high sensitivity [14]. The detection of oncogenic HPV E6 and/or E7 mRNA transcripts using RT-PCR or RT-qPCR is the only method providing direct evidence for a transcriptionally active HPV infection and viral oncogenesis [9,12]. Thus, detection of E6/E7 mRNA in HPV DNA positive cases is often considered as laboratory gold standard, while the clinical gold standard is p16 IHC as detection of E6/E7 mRNA is laborious and prone to sample cross-contamination and thus often not feasible in clinical routine laboratories [12]. All above-mentioned methods require tissue and, consequently, biopsies or surgical resection of tumors [15].

Serum antibodies against HPV16 early proteins, such as the viral oncoproteins E6 and E7, or regulatory proteins E1 and E2, have emerged as promising blood-based biomarkers for the differentiation between HPV-driven and HPV-negative OPC. The measurement of serum antibodies only requires a blood draw, a minimally invasive procedure regularly performed during clinical routine. Detection of serum antibodies against HPV16 early proteins at OPC diagnosis may help to evaluate the need for biopsies in suspected cases and may inform treatment decisions as biomarkers for tumor HPV status where no tumor tissue is available. Moreover, in comparison to tissue-based methods requiring ex-traction and amplification of HPV nucleic acids, serology is less susceptible to sample cross-contamination. In addition, HPV16 early protein serology can be used as a marker for molecular HPV tumor status in epidemiological studies when tissue is not available for the (full) study cohort.

The performance of HPV(16) early protein serology compared to tissue-based reference methods has been addressed in several studies resulting in varying estimates for sensitivity and specificity. This systematic review and meta-analysis aims to summarize the available literature and data in order to increase the precision of sensitivity and specificity estimates, and to explore sources for variability in HPV serology performance.

## 2. Materials and Methods

### 2.1. Inclusion and Exclusion Criteria

The systematic literature review was performed in line with the guidelines provided by the Preferred Reporting Items for Systematic Reviews and Meta-Analyses of Diagnostic Test Accuracy Studies (PRISMA DTA) [16]. Inclusion criteria were as follows: (1) studies published between 2000 and 2020; (2) which included humans with a cancer of the head and neck (HNC) or oropharynx; (3) used at least one reference method to identify HPV-positive tumors (PCR, RT-PCR, ISH or p16 IHC); (4) and reported serology results for HPV16 early proteins using blood collected prior to treatment; (5) as well as information on agreement (2 × 2 tables) between these reference methods and HPV16 serology. Studies reporting oropharyngeal cancers in combination with other head and neck cancers that did not allow separate data extraction were excluded. Reviews, studies including recurrent cancers, or cancer of the head and neck with unknown primary, as well as studies with blood drawn prior to cancer diagnosis or after initiation of cancer treatment were excluded.

### 2.2. Search Strategy and Study Selection

**Figure 1:**
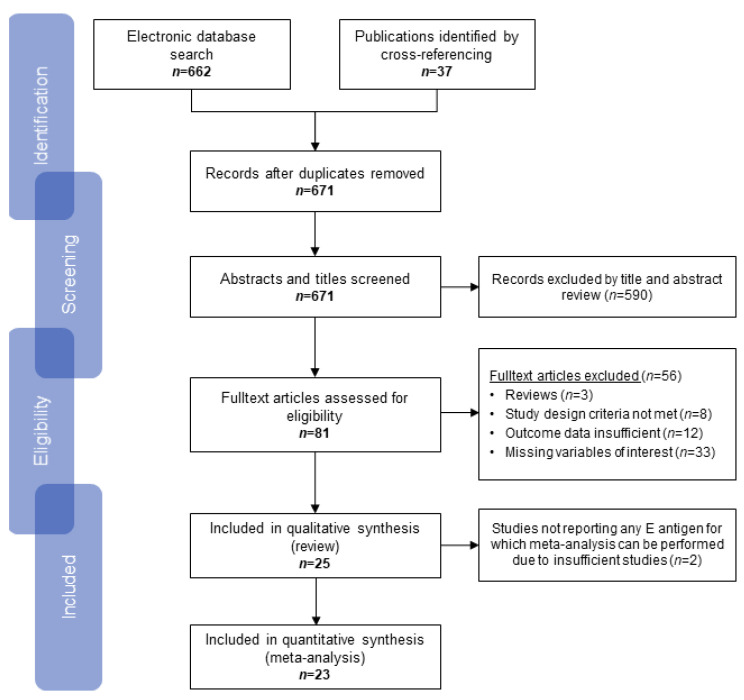
PRISMA flow diagram of study selection process [17].

An electronic literature search was conducted (as of 18 October 2020) in MEDLINE via PubMed and Web of Science to identify relevant studies published from 2000 onwards with the following search term: (oral OR oropharynx OR oropharyngeal OR head and neck) AND (cancer OR neoplasm OR carcinoma) AND (HPV OR human papillomavirus OR human papilloma virus) AND (serolog* OR antibod* OR seropos* OR E6 OR E7) AND (sensitivity OR specificity OR agreement). Additional publications were included by cross-referencing, i.e., we screened the reference lists of included studies for additional relevant studies. No restrictions on language were imposed. Duplicates were removed be-fore screening titles and abstracts according to defined inclusion and exclusion criteria. Afterwards, full-text manuscripts were reviewed for eligibility by two authors (J.H., N.B.). The PRISMA flow diagram [17] is displayed in Figure 1.

### 2.3. Data Extraction and Synthesis

Data was extracted from eligible publications with regard to (1) study characteristics: first author, title, year of publication, number of participants providing samples, countries of origin, cancer type (OPC or HNC); (2) characteristics of reference method and index test, i.e., HPV16 serology used: type of reference method to determine molecular HPV tumor status (HPV DNA PCR, RT-PCR, DNA ISH, RNA ISH, p16 IHC or a combination of those), timeframe of tissue collection, HPV type and antigens used to determine serostatus, type of serologic assay, system used for antigen expression in serologic assays, cut-offs and how they were determined (Tables S1 and S2); (3) for all included early proteins, 2 × 2 table data was extracted or reconstructed (Table S3) in order to calculate sensitivity, specificity and corresponding confidence intervals as well as pooled summary estimates and heterogeneity between studies. Dichotomized data of each serological marker and reference method was extracted as determined by the authors. If studies provided biomarker agreement data on more than one molecular marker, p16 IHC and/or ISH were extracted preferably to HPV DNA PCR only [18]. If more than one study included the same cohort, only the study with the largest sample was subsequently included in the meta-analysis. Reference methods were regrouped into three strata (1) HPV DNA PCR, (2) p16 IHC or ISH and (3) any combination of two reference methods. For studies which did not report values to allow extraction or reconstruction of specificity measurements (true negatives and false positives), only the numbers of true positives and false negatives were extracted as sensitivity and specificity were analyzed independently.

### 2.4. Statistical Analysis

The outcome of interest was defined as the sensitivity and specificity of HPV early protein serology in comparison to established molecular reference methods regarding their ability to distinguish between HPV-driven and HPV-negative OPC. As multiple reference methods for the determination of molecular HPV tumor status in OPC cases have been developed, reference methods were grouped by single or dual positives, and methodical details (histopathological methods vs. PCR). Individual study estimates of sensitivity and specificity were calculated based on extracted 2 × 2 table data with 95% confidence intervals (CI) based on binomial probabilities.

A random effects model was used in each meta-analysis as it accounts for the presence of heterogeneity which is a common occurrence in meta-analysis of diagnostic tests [19]. The inclusion of a wide range of reference methods, serological tests and composition of reported HPV16 early antigens corroborate this assumption. The DerSimonian-Laird procedure was used to fit the random effects model. Pooled summary estimates were calculated using logit-transformed single proportions. CIs were determined using the Clopper-Pearson interval. I^2^ statistics were used to explore and quantify heterogeneity. I^2^ indicates the proportion of variation due to heterogeneity among the total observed variation including random error. Estimates of I^2^ are reported as small (<25%), moderate (25% to 75%) and considerable (>75%). Subgroup and moderator analyses were conducted to explore heterogeneity and identify possible sources of heterogeneity as far as the number of eligible studies allowed. The following characteristics were assessed for HPV16 E2, E6 and E7 serology in meta-analyses based on biological and epidemiological background on OPC, and previous observations of heterogeneity in performance assessment: country, samples size, expression system for HPV16 early antigens, type of reference method, and assay platform. A continuity correction of 0.5 was used in case of zero cells [20]. A risk of bias assessment was performed according to the QUADAS-2 statement [21]. All statistical analyses and graphical illustrations of results were performed in R (Version 4.0.4, packages meta, metafor, tidyverse, gg2plot) and Comprehensive Meta-analysis [22,23,24,25]. Recalculation of estimates using a generalized linear mixed model as sensitivity analysis resulted in almost identical results (median difference = 0.4%).

## 3. Results

### 3.1. Search and Study Selection

The search of electronic databases identified 662 studies and was complemented by cross-referencing adding 37 studies (Figure 1). After removal of duplicates and screening of abstracts, a total of 81 full-text articles were assessed for eligibility according to predetermined in- and exclusion criteria. Publications were excluded due to multiple reasons: reviews (*n* = 3), serum samples were collected before diagnosis (*n* = 8), data on agreement between serology and reference methods was not reported (*n* = 33) or only effect sizes were reported which did not allow to reconstruct sensitivity or specificity estimates (*n* = 12). A total of 25 studies were included in the qualitative synthesis. A total of 23 studies, with study sizes ranging from 10 to 1053 individuals (total number of individuals = 3859), were included in the meta-analyses for HPV16 E6, E7 and E2 serology (Figure 1) [18,26,27,28,29,30,31,32,33,34,35,36,37,38,39,40,41,42,43,44,45,46,47,48,49].

### 3.2. Study Characteristics

The main characteristics of interest extracted from the 25 eligible studies are displayed in Table 1. The full extraction table is shown in Table S1. The majority of the studies included in the meta-analysis used patient samples from North America (*n* = 16), followed by Europe (*n* = 6) and South America (*n* = 1). Twenty-two studies reported serological measurements for HPV16 E6 antibodies, 19 for antibodies against E7, and 11 included E2 antibody measurements. Utilized molecular methods to determine molecular HPV tumor status, i.e., reference methods, included DNA ISH or p16 IHC (*n* = 7), HPV DNA PCR (*n* = 4) and combinations of any two molecular methods (*n* = 12), such as ISH or p16 and PCR or RT-PCR. Proteins used for serology were either expressed in vitro (*n* = 7) or using bacterial expression systems (*n* = 16). Nineteen studies provided complete data to calculate sensitivity and specificity, while four only reported sensitivity measurements [42,43,46,48]. Moreover, six studies relied on cancer-free individuals to estimate the specificity of HPV16 serology [32,34,35,39,45,49]. In these cases, serology results were not compared against HPV-negative tumors as determined by molecular HPV tissue analysis but against cancer-free individuals, i.e., individuals without HPV-driven malignancies. Five of the studies reporting E2 antibody measurements utilized two non-overlapping N (N-E2) and C (C-E2) terminal fragments as the used in vitro expression system did not allow full-length expression of the complete E2 coding sequence due to its length [34,35,36,39,43]. Consequently, these publications contributed two individual E2 fragments to the meta-analysis of E2. All studies using full length E2, further referred to as E2, relied on bacterial expression systems. Serologic methods were either ELISA (*n* = 7) or bead-based suspension arrays (*n* = 16) (Table S1). 

### 3.3. HPV16 E2, E6 and E7 Serology Summary Estimates

**Figure 2:**
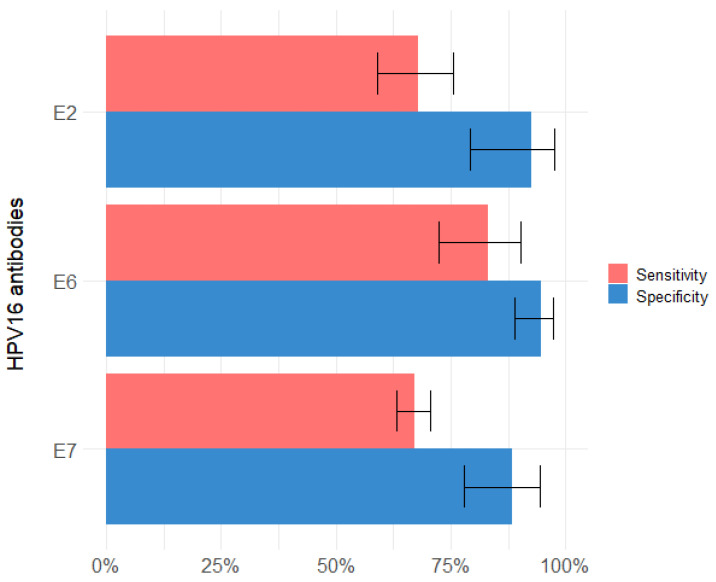
Bar plot of summary estimates of sensitivity (red) and specificity (blue) for HPV16 early protein serology (E2, E6 and E7) in comparison with molecular HPV tumor status. Whiskers represent 95% confidence intervals. Summary estimates were calculated by meta-analyses using random effects models.

The number of studies reporting antibody measurements against single HPV16 early proteins (E1, E2, E4, E5, E6, E7) allowed to perform meta-analyses for E2, E6 and E7 serology. The estimates reported for HPV16 E1, E4 and E5, and combinatorial algorithms for HPV16 serology are summarized in Figures S1–S8. Pooled summary estimates for HPV16 E2, E6 and E7 serology ranged from 67.0% (95% CI 63.2–70.6%) to 83.1% (95% CI 72.5–90.2%) for sensitivity and from 88.5% (95% CI 77.9–94.4%) to 94.6% (95% CI 89.0–97.4%) for specificity (Figure 2).

**Figure 3:**
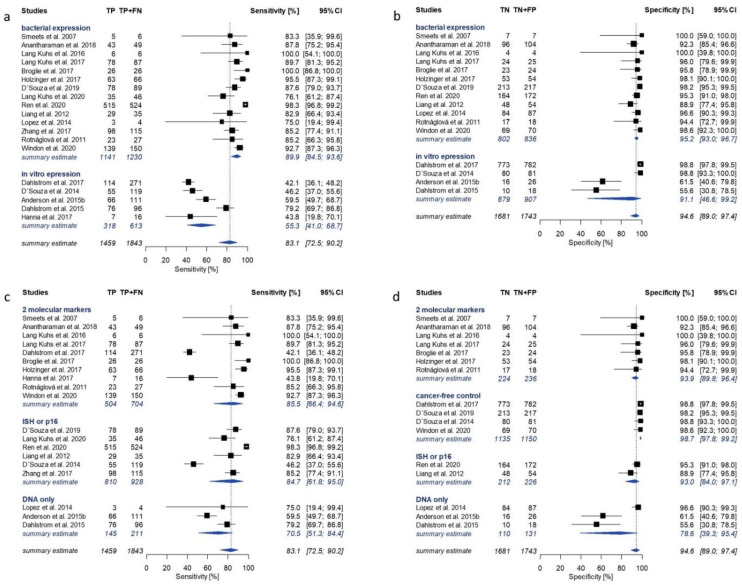
Forest plots of study-specific sensitivity and specificity estimates and summary estimates for HPV16 E6 serology in comparison with molecular HPV tumor status. Pooled estimates were calculated by univariate analysis of log-transformed proportions using a random effects model. Sensitivity of E6 with subgroup summary estimates (I^2^ = 94.4% (92.6–95.8%)) was stratified by (**a**) protein expression system and (**c**) reference method. Specificity of E6 with subgroup summary estimates (I^2^ = 85.5% (77.8–90.5%)) was stratified by (**b**) protein expression system and (**d**) reference method. TP: true positive; FN: false negative; TN: true negative; FP: false positive; CI: confidence intervals; ISH: in situ hybridization; p16: p16 immunohistochemistry; DNA only: HPV DNA PCR.

The highest overall sensitivity of 83.1% (95% CI 72.5–90.2%) and specificity of 94.6% (95% CI 89.0–97.4%) was observed for E6, in a total of 19 and 16 studies, respectively (Figure 2). Individual study reports contributing to pooled estimates for E6 serology ranged from 42.1% to 100% for sensitivity and from 55.6% to 100% for specificity (Figure 3). Be-tween study variance was found to be considerable for both sensitivity (I^2^ = 94.4% (95% CI 92.6–95.8%)) and specificity of E6 (I^2^ = 85.5% (95% CI 77.8–90.5%)).

**Figure 4:**
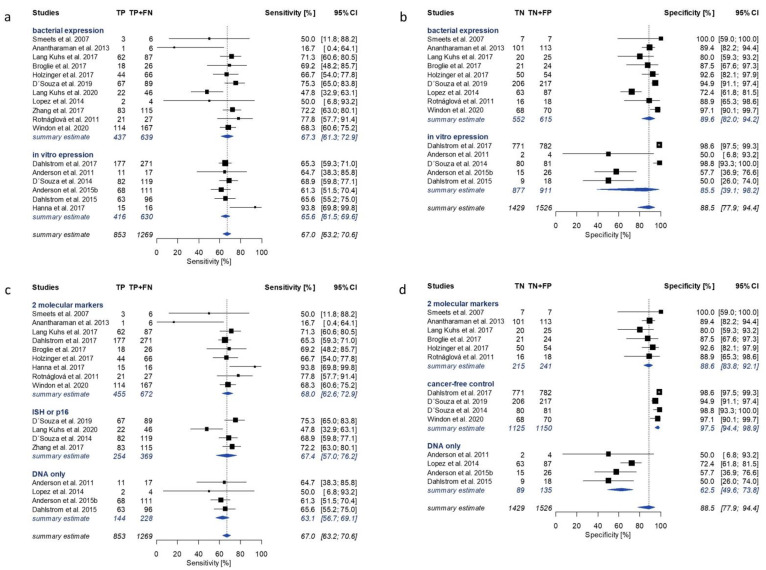
Forest plots of study-specific sensitivity and specificity estimates and summary estimates for HPV16 E7 serology in comparison with molecular HPV tumor status. Pooled estimates were calculated by univariate analysis of log-transformed proportions using a random effects model. Sensitivity of E7 with subgroup summary estimates (I^2^ = 37.1% (0.0–64.8%)) was stratified by (**a**) protein expression system and (**c**) reference method. Specificity of E7 with subgroup summary estimates (I^2^ = 90.4% (85.7–93.6%)) was stratified by (**b**) protein expression system and (**d**) reference method. TP: true positive; FN: false negative; TN: true negative; FP: false positive; CI: confidence intervals; ISH: in situ hybridization; p16: p16 immunohistochemistry; DNA only: HPV DNA PCR.

E7 serology showed both the lowest overall sensitivity of 67.0% (95% CI 63.2–70.6%; 17 studies) and specificity of 88.5% (95% CI 77.9–94.4%; 14 studies; I^2^ = 90.4%) (Figure 2). Single study estimates for sensitivity and specificity of E7 ranged from 16.7% to 93.8%, and from 50% to 100%, respectively (Figure 4). The pooled meta-analysis of the sensitivity of E7 serology did not reveal significant heterogeneity (I^2^ = 37.1% (95% CI 0.0–64.8%)).

**Figure 5:**
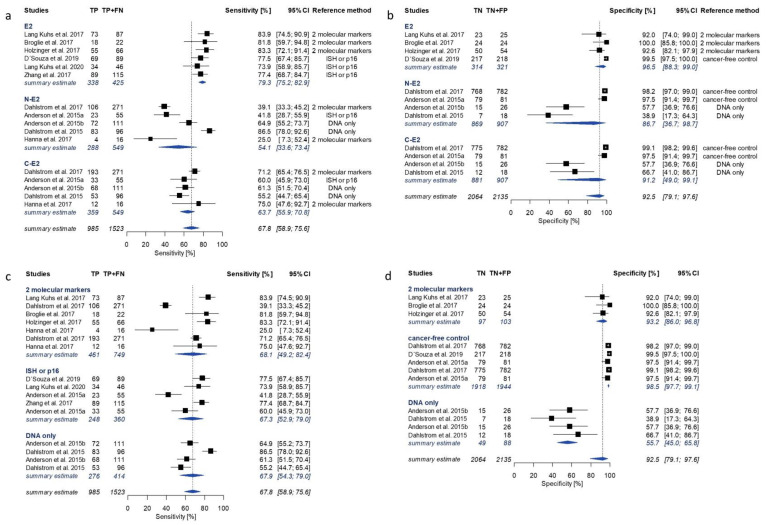
Forest plots of study-specific sensitivity and specificity estimates and summary estimates of HPV16 E2 serology in comparison with molecular HPV tumor status. Pooled estimates were calculated by univariate analysis of log-transformed proportions using a random effects model. Sensitivity of E2 with subgroup summary estimates (I^2^ = 90.6% (86.3–93.5%)) was stratified by (**a**) E2 antigens and (**c**) reference method. Specificity of E2 with subgroup summary estimates (I^2^ = 94.2% (91.6–96.0%)) was stratified by (**b**) protein variant and (**d**) reference method. TP: true positive; FN: false negative; TN: true negative; FP: false positive; CI: confidence intervals; ISH: in situ hybridization; p16: p16 immunohistochemistry; DNA only: HPV DNA PCR.

Pooled study estimates for E2 showed a moderate sensitivity (67.8% (95% CI 58.9–75.6%); 11 studies; I^2^ = 90.6%) and high specificity (92.5% (95% CI 79.1–97.6%); 8 studies; I^2^ = 94.2%) (Figure 2). Subgroup estimates by E2 antigen resulted in higher sensitivity for the full-length E2 antigen (79.3% (95% CI 75.2–82.9%)) when compared to C-E2 (63.7%; 95% CI (55.9–70.8%)) or N-E2 (54.1% (95% CI 33.6–73.4%)) (Figure 5a). Specificity values for all E2 antigens were high (E2: 96.5% (95% CI 88.4–99.0%); C-E2: 91.2% (95% CI 49.0–99.1%); N-E2: 86.7% (95% CI 36.7–98.7%)) (Figure 5b).

### 3.4. Subgroup Analysis

Considerable heterogeneity was observed during the performed meta-analyses for all HPV16 early proteins. In order to explore potential sources of between-study variability and to identify moderators, subgroup analyses were performed for HPV16 E2, E6 and E7 serology based on selected study and assay characteristics. The moderators identified as sources of between-study variability differed for sensitivity and specificity. The most prominent moderator variable for sensitivity was the protein expression system, in contrast to the reference method to determine molecular HPV tumor status for specificity. Figure 3, Figure 4 and Figure 5 show forest plots of HPV16 early antigen serology only for the most influential subgroups. A summary of all investigated moderators can be found in Table S4. Studies utilizing bacterially expressed E6 as antigen reported significantly higher sensitivity of 89.9% (95% CI 84.5–93.6%, 14 studies) compared to those using in vitro expression systems (55.3% (95% CI 41.0–68.7%), 5 studies) (Figure 3a). A similar effect was observed for E2. The subgroup of bacterially expressed E2 showed homogenously high sensitivity (79.3% (95% CI 75.2–82.9%); I^2^ = 0%), while the subgroups of in vitro translated proteins (C-E2 and N-E2) showed lower and more heterogeneous sensitivity estimates (59.5% (95% CI 48.2–69.9%)) (Figure 5a).

Subgroup comparison of specificity of HPV16 early protein serology in comparison with different reference methods showed a distinct pattern for all three HPV16 early proteins. In cancer-free controls, HPV16 early protein serology (E2: 98.5%, E6: 98.7%, E7: 97.5%) performed similarly to studies using a combination of at least two reference methods (E2: 93.2%, E6: 93.9%, E7: 88.6%). In contrast, studies using HPV DNA PCR as reference method showed the lowest specificity for all HPV16 proteins (E2: 55.7%, E6: 78.6%, E7: 62.5%) (Figure 3d, Figure 4d and Figure 5d). This pattern was also observed for HPV16 E1 and E4 serology (Figures S1 and S2).

### 3.5. Other HPV16 Proteins and Combinatorial Algorithms

In addition to the early HPV16 proteins E2, E6 and E7, other early proteins, i.e., E1, E4 and E5, or algorithms combining serological measurements for different HPV16 early proteins to determine overall seropositivity, have been evaluated in comparison with molecular HPV tumor status in some studies. These include, e.g., seropositivity for E6 and/or E7, seropositivity for any early protein, or seropositivity for at least three early proteins. Due to low numbers of studies evaluating these measurements or algorithms, we did not include them in this meta-analysis but summarized the reported performances by study and reference method in the supplement (Figures S1–S8). Reported sensitivities ranged from 1.8% (E5) to 97.0% (seropositivity for any early protein), while specificity ranged from 25.0% (E1) to 100.0% (seropositivity for at least three early proteins; seropositivity for at least three early proteins or E6 > 1000 MFI, seropositivity for E6 and E7; seropositivity for E6 and/or E7).

### 3.6. Risk of Bias Analysis

**Figure 6:**
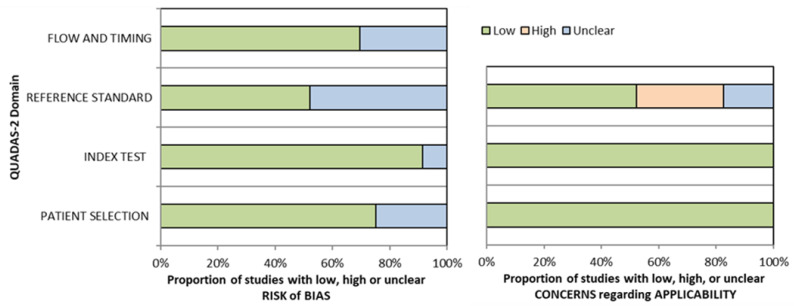
Stacked bar charts of risk of bias assessment according to QUADAS-2 [21]. Study quality expressed as percentage of studies showing low (green), unclear (blue) and high (red) potential of bias in four domains.

Figure 6 displays the determined risk of bias according to the QUADAS-2 assessment tool for studies included in meta-analyses [21]. None of the included studies showed high overall risk of bias. The greatest concern remained applicability of the reference standard, i.e., reference method, domain caused by the use of surrogate markers of active infection as reference standards, such as p16 IHC or HPV DNA PCR.

## 4. Discussion

Antibodies against HPV16 early proteins are considered promising biomarkers for HPV-OPC. Multiple studies evaluated their capability to distinguish between HPV-negative and HPV-driven OPC [50,51]. This systematic review with meta-analysis is a comprehensive assessment of sensitivity and specificity of HPV16 serology for HPV-OPC at the time of diagnosis. We determined summary estimates for the performance of HPV16 E2, E6 and E7 serology prior to treatment in comparison with molecular HPV tumor status and explored potential sources of variation in a total of 23 studies. E6 serology exhibited the best overall performance, with moderately high sensitivity (83.1% (95% CI 72.5–90.2%)) and high specificity (94.6% (95% CI 89.0–97.4%)) when pooling all estimates. Pooled estimates for E2 showed lower sensitivity (67.8% (95% CI 58.9–75.6%)) while maintaining high specificity (92.5% (95% CI 79.1–97.6%)). However, E2 showed diverging performance of sensitivity and specificity among different antigens used (Figure 5). The lowest pooled sensitivity and specificity estimates were observed for E7 serology with similar sensitivity compared to E2 (67.0% (95% CI 63.2–70.6%)), but with a slight decrease in specificity (88.5% (95% CI 77.9–94.4%)).

Even though a subgroup analysis does not provide causal evidence, it can help identify possible sources of variation. We investigated moderating effects of variables which have been shown to introduce heterogeneity in previous studies (reference method, assay platform, protein expression system, region of sample collection and type of study). We explored differences in subgroups separately for sensitivity and specificity. Two moderators affected sensitivity and specificity independently, i.e., the antigen expression system for sensitivity and the reference methods to determine molecular HPV tumor status for specificity (Figure 3, Figure 4 and Figure 5, Table S4).

In comparison with in vitro expression, bacterial expression conferred significantly increased sensitivity for E6 and E2 serology (89.9% vs. 55.3%, and 79.3% vs. 59.5%, respectively) as well as slightly higher specificity (95.2% vs. 91.1%, and 96.5% vs. 86.7–91.2%). The E6 protein was previously reported to be unstable when purified, and utilizing the E6 protein in its native conformation in serological assays is challenging [52]. Protein expression and purification conditions can impact protein folding and conformational stability in serological assays. Thus, both the expression system and purification conditions may affect the antibody binding capacity of the E6 protein, probably resulting in diverging sensitivity of E6 serology in different serological assays. Studies including E2 estimates utilized different versions of the protein for antibody detection, i.e., those using in vitro expression systems used fragmented proteins due to poor translation of the full-length sequence [29] while those using bacterial expression systems used the full-length protein. Even though in vitro expressed C-E2 was more sensitive than N-E2 (63.7% and 54.1%, respectively), both exhibited lower sensitivity and higher variation between single studies than bacterially expressed full-length E2 (sensitivity 79.3%). This suggests that at least a portion of the serum antibodies against E2 in HPV-OPC cases may not be able to bind to the fragmented proteins due to conformational differences between the presented epitopes of the native full-length protein and the expressed fragments. In addition to the fragmentation, the expression system or purification conditions may also impair the antibody binding capacity of C-E2 and N-E2. Unlike E2 and E6, E7 serology showed lower between study variation for sensitivity (I^2^ = 37.1%), especially among those studies including substantial numbers of OPC cases, and was hardly impacted by the investigated moderators.

The subgroup analysis further revealed a particular pattern in specificity of HPV16 early antigen serology by reference method. In most studies relying on HPV DNA PCR alone as reference method, HPV16 early antigen serology showed significantly lower specificity compared to those studies using histopathological reference methods or combinations of two reference methods. This contributed substantially to the observed heterogeneity of the summary estimates and was observed for all early proteins included in the meta-analyses, and for HPV16 E1 and E4 (Figure 3, Figure 4 and Figure 5d, Figures S1 and S2). Among the three studies using HPV DNA PCR as reference method, only Lopez et al. showed high specificity for E6 and E7 serology [33,35,36]. The studies using HPV DNA PCR as a reference method used different primers affecting test accuracy of the reference method (Table S1), as the HPV DNA PCR method used by Lopez et al. showed superior sensitivity compared to the PCR methods utilized by the other studies in this subgroup [53,54]. Thus, the less sensitive PCR methods may have missed some true positives detected by HPV16 early protein serology, which were then wrongly classified as false positives. In addition, the probability of misclassifying a negative case is already much lower when the proportion of HPV-attributable cases among OPCs is very low, as is the case in Brazil with only 4% compared to the United States with over 70% [2,3]. Lower sensitivity of HPV16 E6 serology in the subgroup of studies using HPV DNA PCR to determine molecular HPV tumor status is probably based on a combination of lower specificity of HPV DNA PCR for detection of HPV-OPC due to (i) potential sample-cross contamination [55] and (ii) a lack of evidence for the functional involvement of HPV when merely detecting DNA [12,56], and the lower sensitivity of in vitro expressed E6 antigen.

In clinical practice, p16 IHC is often used as a surrogate marker for defining molecular HPV tumor status. However, p16 overexpression may not only be induced during HPV-driven carcinogenesis but can also be mediated by non-HPV-related mechanisms during carcinogenesis, thus generally limiting the specificity of p16 IHC for HPV-OPC status (approximately 83%) [12]. Assuming reduced specificity of the reference method, i.e., the detection of a proportion of false positives, we would have expected to observe a lower sensitivity of HPV16 early protein serology in comparison with p16 IHC. However, we did not observe a systematic difference in sensitivity of HPV16 early protein serology in the subgroup of studies using one histopathological reference method, i.e., p16 IHC or ISH in contrast with studies using combinations of molecular methods to determine molecular HPV tumor status. The three studies using p16 IHC only as a reference method were Lang Kuhs et al. 2020, Ren et al. 2020 and Liang et al. 2012, and the sensitivities for HPV16 E6 serology ranged between 82.9% and 98.3% in contrast with a summary estimate of 85.5% (range 42.1–100.0%) for studies using two molecular markers [18,46,47]. The three p16 IHC studies were conducted in North America, a region of high HPV-AF probably explaining the non-superior performance of two molecular markers in contrast to p16 IHC only. In regions with lower HPV-AF for OPC, p16 IHC may detect more false-positives and would also impair the sensitivity estimate of HPV16 serology in a comparative study. The only study in the single histopathological marker group with lower sensitivity estimate for HPV16 E6 serology was D’Souza et al. 2014 [32] which is also the only study in this group using in vitro expressed antigens in comparison with bacterially expressed antigens, probably explaining the lower reported sensitivity.

This systematic review included case–control, case–case as well as case-only studies. Although these different study designs lead to similar outcomes in HPV16 serology performance, the nature of the selected controls affects the interpretation of the resulting specificity estimates. A case–case study aims to distinguish whether an OPC case is HPV-related or not by comparing tissue and blood samples of patients with oropharyngeal cancer. Studies using cancer-free controls aim to investigate the potential of HPV16 early serology to differentiate between individuals with HPV-driven OPC and healthy, cancer-free individuals. The subgroup estimates showed very high specificity of HPV16 serology in cancer-free controls and all reference methods with the exception of HPV DNA PCR. The specificity estimates are comparably high across different study designs because antibodies against HPV16 early proteins are rare among individuals of the general population and individuals without HPV-associated malignancies (e.g., [31,57]). Methodologically, case–control-based studies are more susceptible to bias due to the selection of controls from a different population and less applicable to clinical settings. We assessed potential bias caused by independent controls but did not find significant variation by study design (Table S5) [58].

A recent review and meta-analysis by Balachandra et al. investigated blood-based biomarkers for the identification of HPV-driven cancers including HPV16 E6 serology for oropharyngeal cancer [59]. After exclusion of case–control and case-only studies, they identified three studies reporting HPV16 E6 serology in comparison with a molecular reference method (two of those were also included in this review [34,35]). Balachandra et al. reported pooled summary estimates of 56.3% (95%CI 44.4–68.1%) and 99% (95%CI 98.2–99.7%) for HPV16 E6 sensitivity and specificity, respectively. The comparability with this meta-analysis is limited due to differential exclusion criteria leading to an exclusion of most studies by Balachandra et al. we considered relevant in this work. Because we included all study designs in this meta-analysis, our estimates provide a more accurate representation of the published literature on the performance of HPV16 serology, and allowed for exploration of moderators. We showed that sensitivity of HPV16 E6 serology is dependent on the expression system and increases to 89.9% for the assays using bacterially expressed E6. Balachandra et al. further included different combinations of early protein panels that showed higher accuracy than single proteins in their review (e.g., E6 and/or E7, ≥3 early protein antibodies). Although single studies have reported promising results for several panel combinations, we excluded them from this systematic review and meta-analysis due to the very limited number of published studies investigating these combinations [35,41,45,49]. For completeness, we summarized the estimates reported in the available literature in Figures S4–S8. Balachandra et al. further reported on cell-free HPV DNA (cfHPV DNA), another emerging tissue-independent biomarker for the determination of molecular HPV tumor status in OPC and post-treatment surveillance. cfHPV DNA has been reported to demonstrate high sensitivity and specificity to determine molecular tumor HPV status in OPC [60], and its performance warrants comparison against HPV16 early antigen serology in future studies.

This systematic literature review presents a comprehensive overview of the published literature on HPV16 serology performance for several early antigens to determine molecular HPV tumor status among OPC patients in comparison with tissue-based reference methods. However, there are also some limitations. First, there was substantial heterogeneity between the included studies, potentially limiting the precision of the derived sensitivity and specificity estimates and the power of the performed analysis. However, this was expected as a common occurrence in diagnostic test accuracy studies. Second, studies which reported serology results and their agreement with reference methods only as validation subsets often featured very small sample sizes which can increase uncertainty. However, our sensitivity analysis did not identify small sample size as a source of bias. Third, although the detection of HPV mRNA is nowadays considered the laboratory gold standard for the diagnosis of HPV-OPC, only very few studies utilized this reference method; most relied on less reliable reference methods such as HPV DNA PCR, p16 IHC and DNA ISH. Systematic misclassification of samples by less sensitive and/or specific reference methods may have resulted in higher type I and type II error rates, and may have caused over- or under-estimation of serology performance. Indeed, stratification of studies according to the used reference method suggested higher error rates caused by HPV DNA PCR than any other reference method. Lastly, the differences in study designs restrict the generalizability of our specificity estimates and suggest independent analyses in cancer-free controls in the future.

In this systematic literature review, we provided summary estimates and further identified the expression system and reference methods as significant moderators for HPV16 serology performance. This shows that the performance of HPV16 early protein serology has to be evaluated in the context of the characteristics of the reference method and the setting in which it was applied, e.g., a region/study with high or low HPV-AF. The preparation of this systematic review further revealed the lack of available information on the performance of HPV serology for other oncogenic HPV types, and serological HPV(16) biomarker combinations. The identification of parameters associated with performance of HPV early antigen serology may facilitate further method optimization in the future.

## 5. Conclusions

In this systematic review and meta-analysis, we provide robust evidence that antibodies against HPV16 early proteins, especially HPV16 E6, are tumor-tissue-independent and highly sensitive and specific biomarkers for the detection of HPV-driven OPC at diagnosis.

## Figures and Tables

**Table 1:** Summary table of main characteristics for all studies included in the systematic review (*n* = 25) and meta-analysis (*n* = 23) sorted by year of publication.

First Author	Year	*n* Total	HPV+ (*n*)	HPV− (*n*)	Reference Method(s)	Antigen Expression System	HPV16 Antigens ^1^	Country of Origin	Time of Collection ^2^
Herrero ^3^ [26]	2003	137	26	111	DNA PCR	bacterial	E6 and/or E7	Italy, Spain, Ireland, Poland, India, Cuba, Sudan, Canada, Australia	1996–1999
Smeets [27]	2007	13	6	7	RT-PCR and DNA PCR	bacterial	E6, E7	Netherlands	na
D’Souza ^3^ [28]	2010	119	85	34	DNA ISH	bacterial	E6 and/or E7	USA	2000–2006
Anderson [29]	2011	21	17	4	DNA PCR	in vitro	E7, E1	USA	na
Rotnáglová [30]	2011	45	27	18	DNA PCR and RT-PCR	bacterial	E6, E7	Czech Republic	2001–2007
Liang [18]	2012	89	35	54	p16 IHC	bacterial	E6	USA	1999–2003
Anantharaman ^4^ [31]	2013	119	6	113	p16 IHC and DNA PCR	bacterial	E6, E7	Germany, Greece, Italy, Ireland, UK, Spain, Norway, Czech Republic, Croatia	2000–2005
D’Souza ^4^ [32]	2014	200	119	81 ^5^	p16 IHC and/or ISH ^6^	in vitro	E6, E7	USA	2009–2013
Lopez [33]	2014	91	4	87	DNA PCR	bacterial	E6, E7	Brazil	1998–2008
Anderson ^4^ [34]	2015a	136	55	81 ^5^	p16 IHC and/or ISH ^6^	in vitro	E6, E7, E1, E2, E4, E5	USA	2009–2013
Anderson [35]	2015b	137	111	26 ^5^	DNA PCR	in vitro	E6, E7, E1, E2, E4, E5	USA	2006–2008
Dahlstrom [36]	2015	114	96	18	DNA PCR	in vitro	E6, E7, E1, E2, E4, E5	USA	2006–2008
Lang Kuhs [37]	2016	10	6	4	p16 IHC and ISH	bacterial	E6	USA	2003–2006
Lang Kuhs [38]	2017	112	87	25	p16 IHC and ISH	bacterial	E6, E7, E1, E2, E4	USA	2000–2017
Dahlstrom [39]	2017	1053	271	782 ^5^	p16 IHC and ISH	in vitro	E6, E7, E1, E2, E4, E5	USA	2003–2013
Broglie [40]	2017	50	26	24	p16 IHC and DNA PCR	bacterial	E6, E7, E1, E2, E4	Switzerland	na
Holzinger [41]	2017	120	66	54	(NASBA or RT-PCR) and DNA PCR	bacterial	E6, E7, E1, E2	Italy, Germany	na
Zhang [42]	2017	115	115	- ^7^	p16 IHC and/or ISH ^6^	bacterial	E6, E7, E1, E2, E4	USA	2009–2013
Hanna [43]	2017	16	16	- ^7^	p16 IHC and (ISH or DNA PCR)	in vitro	E6, E7, E2	USA	2013–2015
Anantharaman ^4^ [44]	2018	153	49	104	p16 IHC and DNA PCR	bacterial	E6	Germany, Greece, Italy, Ireland, UK, Spain, Norway, Czech Republic, Croatia	2002–2005
D’Souza [45]	2019	306	89	217 ^5^	p16 IHC and/or ISH ^6^	bacterial	E6, E7, E1, E2	USA	2009–2013
Lang Kuhs [46]	2020	46	46	- ^7^	p16 IHC	bacterial	E6, E7, E1, E2, E4	USA	2017–2018
Ren [47]	2020	696	524	172	p16 IHC	bacterial	E6	Canada	na
Fakhry ^4^ [48]	2020	146	146	- ^7^	p16 IHC and RNA ISH	bacterial	E6, E7	USA	2013–2018
Windon ^4^ [49]	2020	220	150	70 ^5^	p16 IHC and RNA ISH	bacterial	E6, E7	USA	2013–2018

ISH: in situ hybridization; IHC: immunohistochemistry; NASBA: nucleic acid sequence-based amplification assay; HPV+: HPV-driven; HPV−: HPV-negative; na: not available; ^1^ HPV16 antigens corresponding to antibodies detected by serology. ^2^ Recruitment of study participants and collection of tissue and blood samples. ^3^ Studies included in review but not meta-analysis. ^4^ Samples also included in another study. For the meta-analysis, only the larger sample size was used. ^5^ population-based cancer-free controls. ^6^ Some individuals may have been tested by p16 IHC and DNA ISH. ^7^ No information on HPV-negative OPC (true negatives and false positives) was reported. Thus, it was not possible to calculate specificity estimates.

## Data Availability

Data is contained within the article and Supplementary Materials.

## Supplementary Materials

The following are available online at https://www.mdpi.com/article/10.3390/cancers13123010/s1, Figure S1: Overview on sensitivity and specificity of HPV16 E1 serology, Figure S2: Overview on sensitivity and specificity of HPV16 E4 serology, Figure S3: Overview on sensitivity and specificity of HPV16 E5 serology, Figure S4: Overview on sensitivity and specificity of combined HPV16 E6 and/or E7 serology, Figure S5: Overview on sensitivity and specificity of combined HPV16 E6 and E7 serology, Figure S6: Overview on sensitivity and specificity of HPV16 serology positive for any early protein, Figure S7: Overview on sensitivity and specificity of HPV16 serology positive for 3 or more early proteins, Figure S8: Overview on sensitivity and specificity of HPV16 serology positive according to individual algorithms. Table S1: Extraction table for all included studies; Table S2: Summary of expression system for HPV16 antigens and corresponding serological assays as described in the included papers; Table S3: Extraction table of reported true positives, true negatives, false positives and false negatives of HPV16 early antigen serology in comparison with molecular tumor HPV status in included OPC cases for calculation of sensitivity, specificity and corresponding 95% confidence intervals.; Table S4: Subgroup analysis of HPV16 E2, E6 and E7 serology, Table S5: Sensitivity analysis.
