# Supplementary material for: Sensitivity and Specificity of Human Papillomavirus (HPV) 16 Early Antigen Serology for HPV-Driven Oropharyngeal Cancer: A Systematic Literature Review and Meta-Analysis
Source: Cancers (Basel). 2021 Jun 16;13(12):3010. doi: 10.3390/cancers13123010 (PMC8234521; doi:10.3390/cancers13123010)
Supplement: Supplementary file 1 [file cancers-13-03010-s001.zip › cancers-1238460-supplementary.pdf]

Supplementary Materials: Sensitivity and Specificity of Human Papillomavirus (HPV) 16 Early Antigen Serology for HPV-Driven Oropharyngeal Cancer: A Systematic Literature Review and Meta-Analysis

Julia Hibbert, Gordana Halec, Dan Baaken, Tim Waterboer and Nicole Brenner

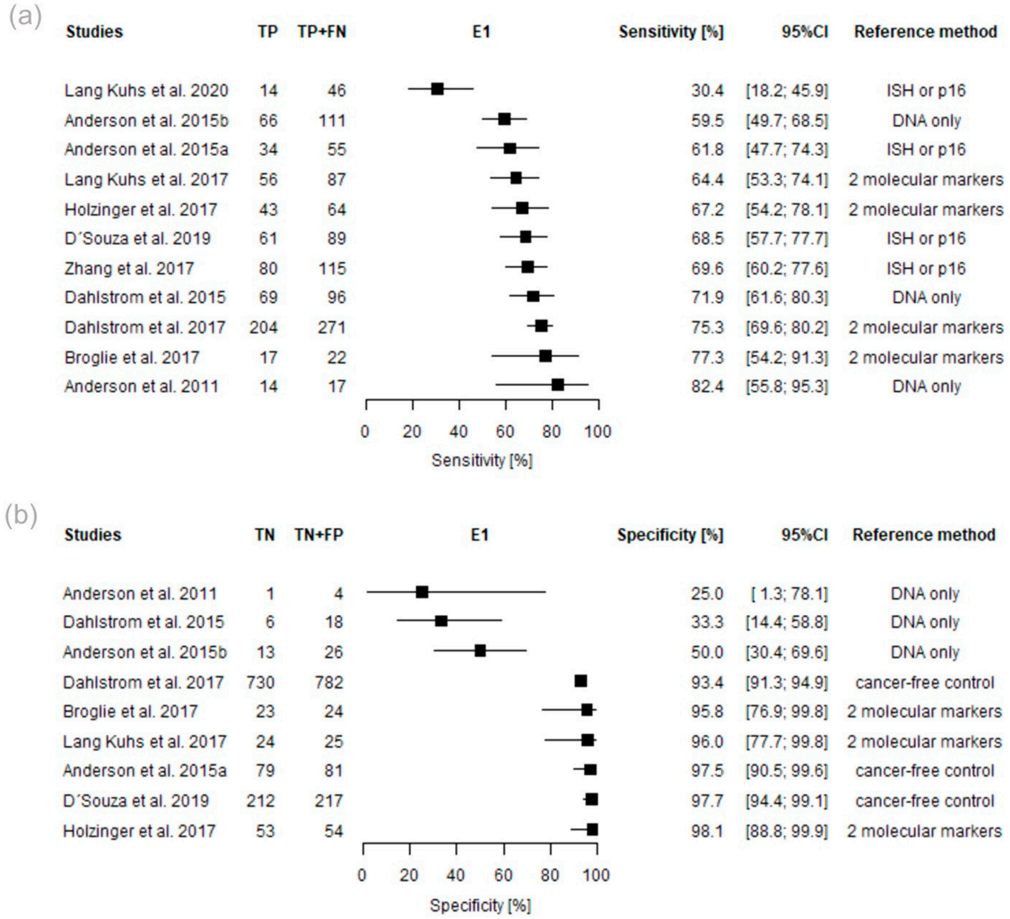

Figure S1. Overview on sensitivity (**a**) and specificity (**b**) of HPV16 E1 serology in comparison with molecular HPV tumor status. TP: true positive; FN: false negative; TN: true negative; FP: Figure 16. p16 immunohistochemistry, DNA only: HPV DNA PCR.

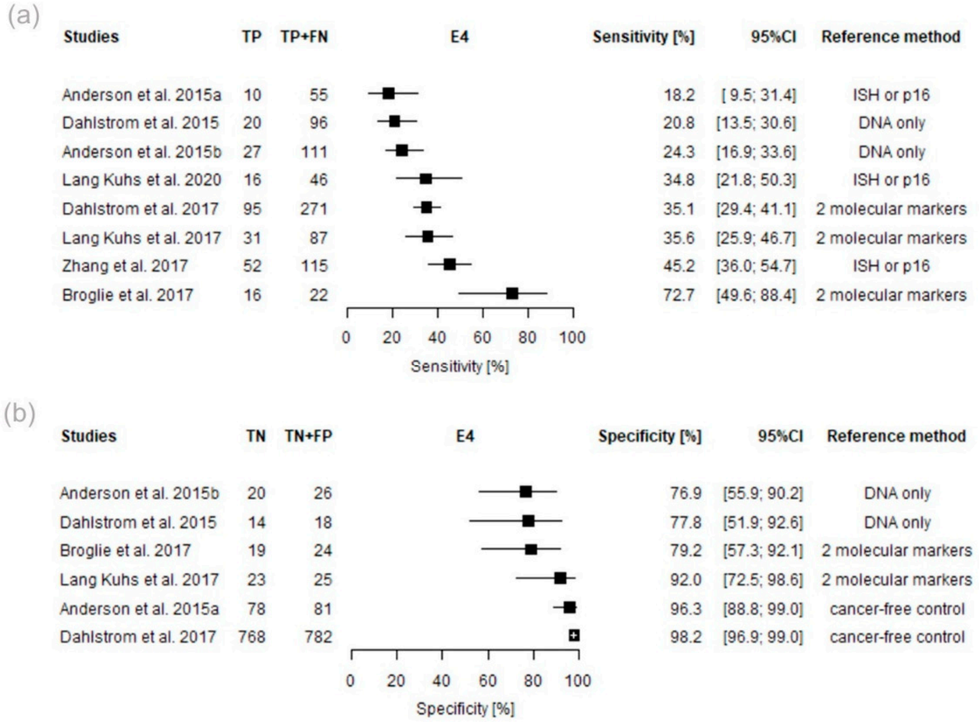

Figure S2. Overview on sensitivity (**a**) and specificity (**b**) of HPV16 E4 serology in comparison with molecular HPV tumor status. TP: true positive; FN: false negative; TN: true negative; FP: false positive; CI: confidence intervals; ISH: in situ hybridization; p16: p16 immunohistochemistry, DNA only: HPV DNA PCR.

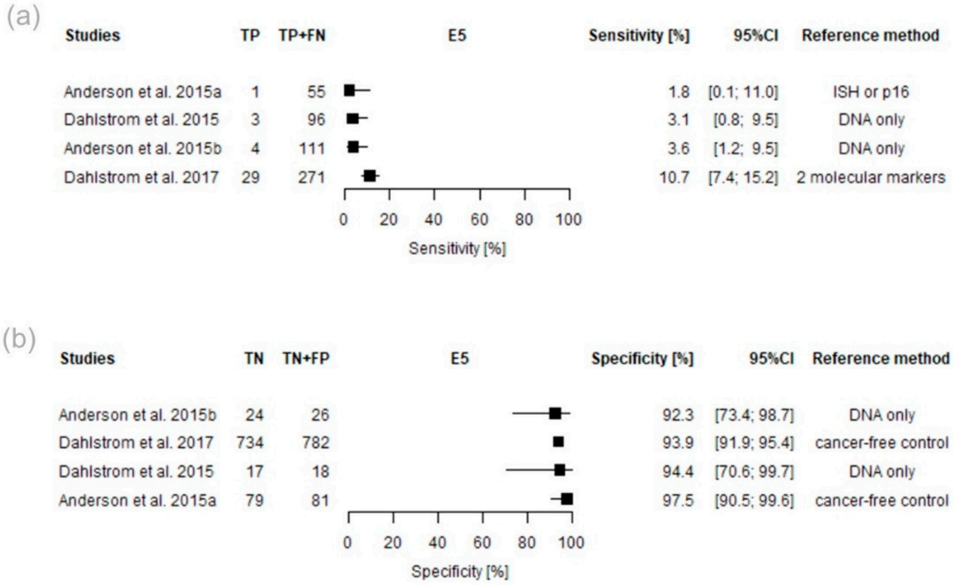

Figure S3. Overview on sensitivity (**a**) and specificity (**b**) of HPV16 E5 serology in comparison with molecular HPV tumor status. TP: true positive; FN: false negative; TN: true negative; FP: false positive; CI: confidence intervals; ISH: in situ hybridization; p16: p16 immunohistochemistry, DNA only: HPV DNA PCR.

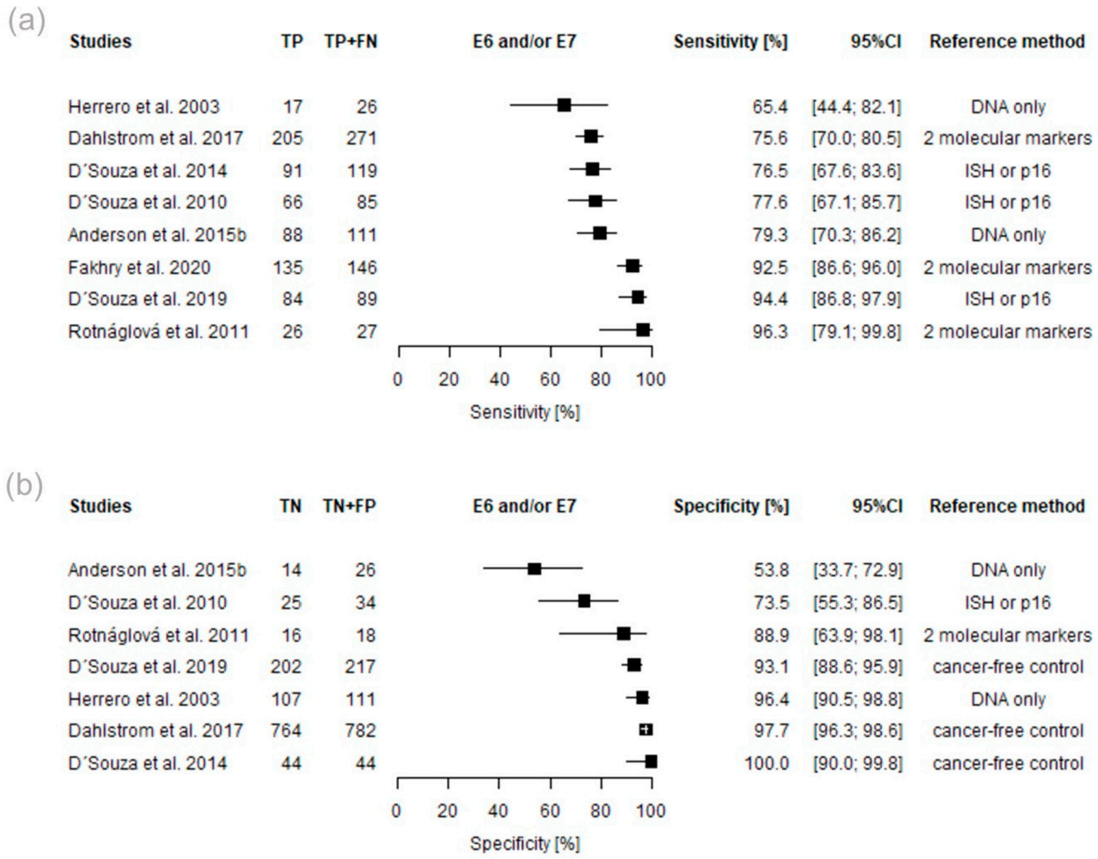

Figure S4. Overview on sensitivity (**a**) and specificity (**b**) of combined HPV16 E6 and/or E7 serology in comparison with molecular HPV tumor status. TP: true positive; FN: false negative; TN: true negative; FP: false positive; CI: confidence intervals; ISH: in situ hybridization; p16: p16 immunohistochemistry, DNA only: HPV DNA PCR.

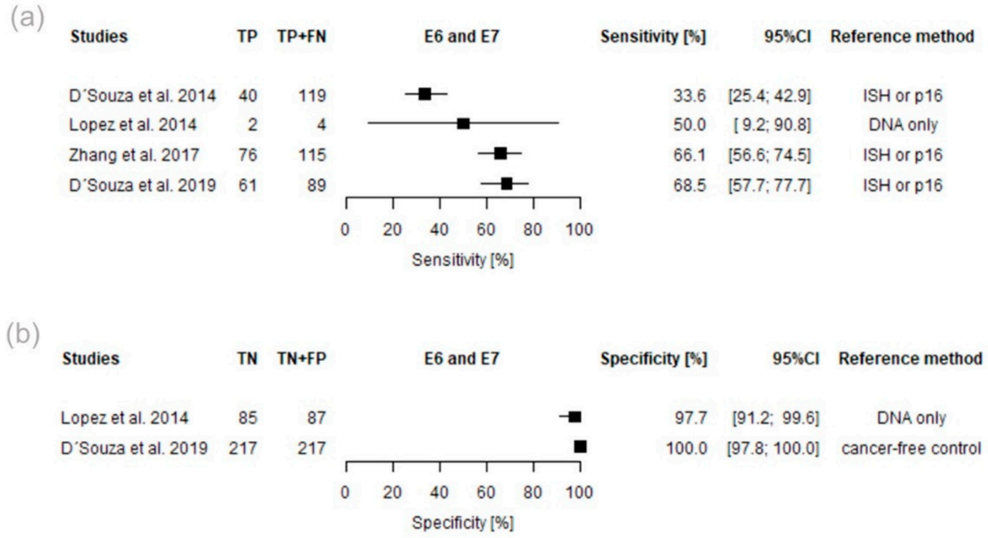

Figure S5. Overview on sensitivity (**a**) and specificity (**b**) of combined HPV16 E6 and E7 serology in comparison with molecular HPV tumor status. TP: true positive; FN: false negative; TN: true negative; FP: false positive; CI: confidence intervals; ISH: in situ hybridization; p16: p16 immunohistochemistry, DNA only: HPV DNA PCR.

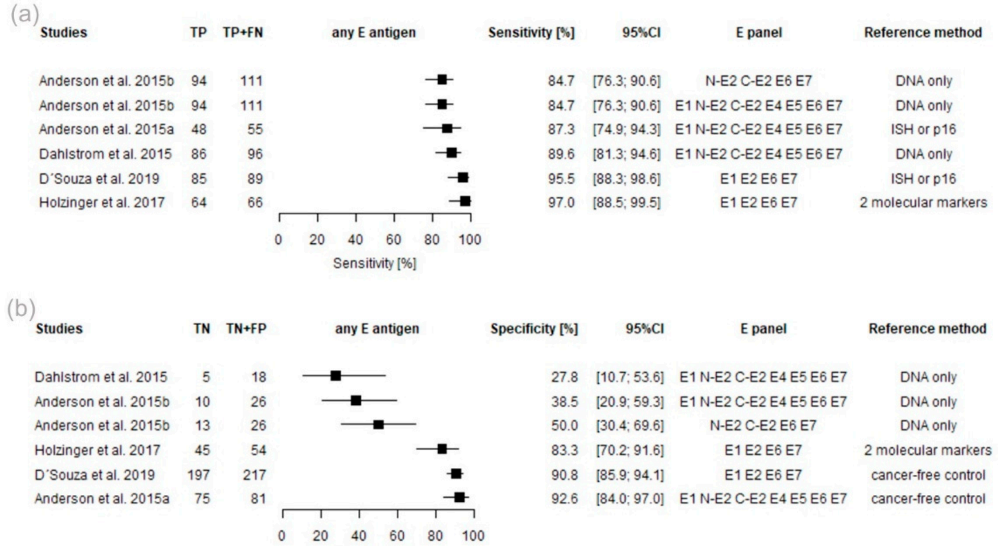

Figure S6. Overview on sensitivity (**a**) and specificity (**b**) of HPV16 protein serology determining positivity by seropositivity against any measured early protein in comparison with molecular HPV tumor status. TP: true positive; FN: false negative; TN: true negative; FP: false positive; CI: confidence intervals; ISH: in situ hybridization; p16: p16 immunohistochemistry, DNA only: HPV DNA PCR.

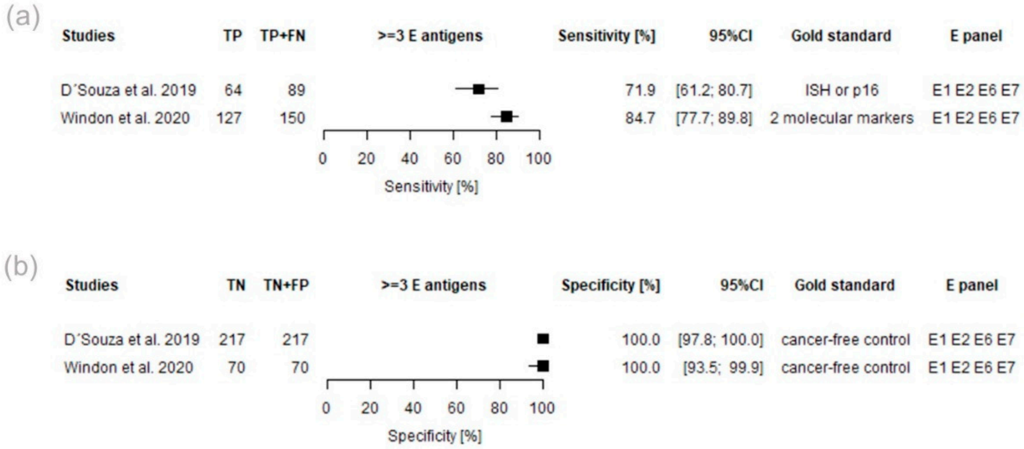

Figure S7. Overview on sensitivity (**a**) and specificity (**b**) of HPV16 early protein serology defining positivity by seropositivity against at least three (E1, E2, E6, E7) early proteins in comparison with molecular HPV tumor status. TP: true positive; FN: false negative; TN: true negative; FP: false positive; CI: confidence intervals; ISH: in situ hybridization; p16: p16 immunohistochemistry.

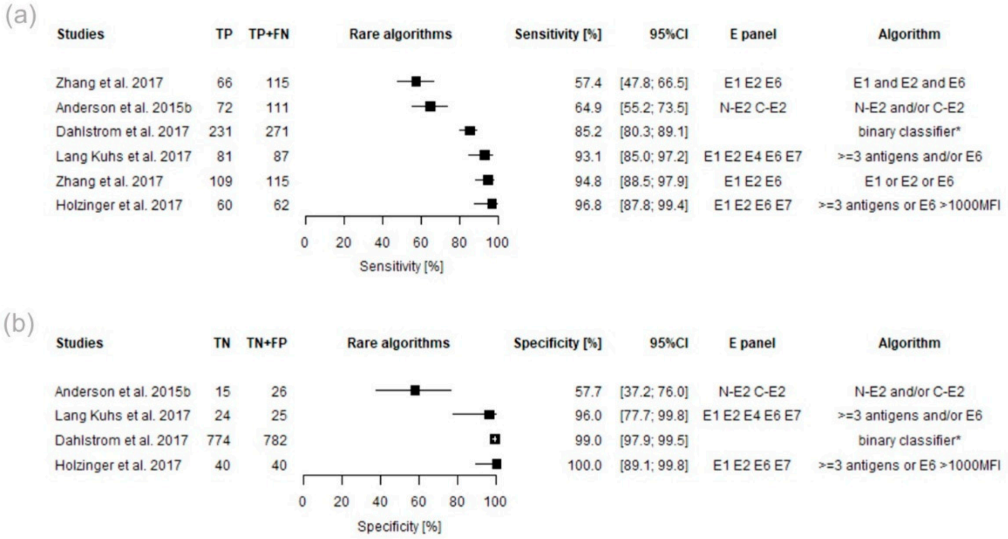

Figure S8. Overview on sensitivity (**a**) and specificity (**b**) of HPV16 early protein serology defining positivity by rare algorithms used in single studies in comparison with molecular HPV tumor status. *The binary classifier uses a two-class logistic regression model based on the serological response against E1, NE2, CE2, E4, E5, E6 and E7. Reference methods: HPV DNA PCR (Anderson et al. 2015b); in situ hybridization or p16 immunohistochemistry (Zhang et al. 2017); 2 molecular markers (Dahlstrom et al. 2017, Holzinger et al. 2017, Lang Kuhs et al. 2017). TP: true positive; FN: false negative; TN: true negative; FP: false positive; CI: confidence intervals.

Table S4. Subgroup analysis for HPV16 E2, E6 and E7 serology in comparison with molecular HPV tumor status. Meta-analyses were performed using random effects models.

	Subgroup		k	Sensitivity [%] (95% CI)	I^2[%]	k	Specificity [%] (95% CI)	I^2 [%]
	overall summary estimate		16	67.8 (58.9–75.6)	90.6	12	92.5 (79.1–97.6)	94.2
	protein variant	E2	6	79.3 (75.2–82.9)	0.0	4	96.5 (88.3–99.0)	59.6
		C-terminal E2	5	63.7 (55.9–78)	62.0	4	91.2 (49.0–99.1)	96.2
		N-terminal E2	5	54.1 (33.6–73.4)	93.9	4	86.7 (36.7–98.7)	97.0
	expression system	bacterial	6	79.3 (75.2–82.9)	0.0	4	96.5 (88.3–99.0)	59.6
		in vitro	10	59.5 (48.2–69.9)	91.0	8	89.1 (64.0–97.4)	96.1
E2	region	Europe	2	na	na	2	na	na
		North America	14	65.6 (56.1–74.1)	91.0	10	91.8 (74.5–97.8)	95.2
		South America	0	na	na	0	na	na
	reference method	2 molecular markers	7	68.1 (49.2–82.4)	94.2	3	93.2 (86.0–96.8)	0.0
		ISH or p16	5	67.3 (52.9–79.0)	84.8	0	na	na
		HPV DNA PCR	4	67.9 (54.3–79.0)	86.2	4	55.7 (45.0–65.8)	0.0
		cancer-free controls	0	na	na	5	98.5 (97.7–99.1)	16.0
	date of publication	before 2015	6	62.8 (51.1–73.1)	84.5	6	78.0 (52.2–92.0)	88.1
		after 2015	10	79.0 (57.5–81.4)	92.8	6	97.8 (94.9–99.0)	71.8

	assay platform	ELISA	8	55.6 (43.5–67.1)	90.2	6	94.1 (74.3–98.9)	96.1
		bead-based	8	77.9 (69.7–84.4)	78.3	6	92.0 (66.0–97.7)	88.1
E6	overall summary estimate		19	83.1 (72.5–92)	94.4	16	94.6 (89.0–97.4)	85.5
	expression system	bacterial	14	89.9 (84.5–93.6)	74.8	12	95.2 (93.0–96.7)	17.0
		in vitro	5	55.3 (41.0–68.7)	89.9	4	91.1 (46.6–99.2)	96.5
	region	Europe	5	93.0 (83.2–94.6)	17.4	5	93.8 (89.3–96.5)	0.0
		North America	13	80.0 (65.9–89.2)	95.9	10	94.1 (84.0–98.0)	91.0
		South America	1	na	na	1	na	na
	reference method	2 molecular markers	10	85.5 (66.4–94.6)	94.2	7	93.9 (89.8–96.4)	0.0
		ISH or p16	6	84.7 (61.8–95.0)	96.3	2	na	na
		HPV DNA PCR	3	75.0 (51.3–84.4)	78.1	3	78.6 (39.3–95.4)	90.1
		cancer-free controls	0	na	na	4	98.7 (97.8–99.2)	0.0
	date of publication	before 2015	7	72.0 (57.4–83.1)	83.5	7	89.2 (72.5–96.3)	83.2
		after 2015	12	87.6 (72.7–94.9)	96.0	9	97.0 (94.4–98.4)	60.1
	assay platform	ELISA	6	55.2 (43.4–66.5)	78.8	5	95.0 (68.7–99.4)	93.3
		bead-based	13	89.6 (83.8–93.5)	80.2	11	94.3 (88.7–97.2)	76.3
E7	overall summary estimate		17	67.0 (63.2–76.0)	37.1%	14	88.5 (77.9–94.4)	90.4
	expression system	bacterial	11	67.3 (61.3–72.9)	47.0	9	89.6 (82.0–94.2)	77.0
		in vitro	6	65.6 (61.5–69.6)	9.1	5	85.5 (39.1–98.2)	96.0
	region	Europe	5	65.4 (51.9–76.9)	40.1	5	89.9 (85.1–93.3)	0.0
		North America	11	67.2 (63.0–71.1)	45.2	8	89.0 (68.6–96.8)	93.6
		South America	1	na	na	1	na	na
	reference method	2 molecular markers	9	68.0 (62.6–72.9)	30.7	6	88.6 (83.8–92.1)	0.0
		ISH or p16	6	67.4 (57.0–76.2)	73.1	0	na	na
		HPV DNA PCR	6	63.1 (56.7–69.1)	0.0	4	62.5 (49.6–73.8)	37.2
		cancer-free controls	0	na	na	4	97.5 (94.4–98.9)	69.6
	date of publication	before 2015	8	65.0 (58.9–76.0)	17.3	8	79.5 (63.4–89.6)	80.3
		after 2015	9	68.2 (63.1–72.8)	50.4	6	94.2 (86.8–97.6)	84.1
	assay platform	ELISA	6	66.7 (64.0–72.5)	36.0	5	93.8 (65.3–99.2)	93.9

bead-based	11	66.9 (61.7–71.8)	41.9	9	85.2 (73.6–92.3)	84.8

k = number of studies within subgroup; na = insufficient data to calculate estimates, i.e., less than three studies or not possible due to study design.

Table S5. Sensitivity analysis excluding studies based on their study design and outliers. None of the studies conferred a high risk of bias (see Figure 6). Thus, no sensitivity analysis was conducted excluding studies based on their risk of bias.

	Omitted Subgroup	k	Sensitivity [%] (95% CI)	I^2 [%] (95% CI)	k	Specificity [%] (95% CI)	I^2 [%] (95% CI)
**E2**	overall summary estimate	16	67.8 (58.9–75.6)	90.6 (86.3–93.5)	12	92.5 (79.1–97.6)	94.2 (91.6–96.0)
	cancer-free controls	7	79.3 (64.1–89.1)	87.4 (72.9–94.1)	5	83.1 (54.8–95.2)	84.4 (65.0–93.0)
	small studies (<25)	14	69.4 (60.3–77.2)	91.3 (87.2–94.1)	12	92.5 (79.1–97.6)	94.2 (91.6–96.0)
	outliers	12	71.8 (66.1–76.9)	71.0 (47.9–83.9)	8	96.0 (89.4–98.6)	83.5 (69.1–91.2)
**E6**	overall summary estimate	19	83.1 (72.5–90.2)	94.4 (92.6–95.8)	16	94.6 (89.0–97.4)	85.5 (77.8–90.5)
	cancer-free controls	11	90.4 (82.4–95.0)	79.9 (64.8–88.5)	11	92.7 (86.3–96.2)	67.6 (39.1–82.8)
	small studies (<25)	15	84.6 (73.4–91.6)	95.6 (94.0–96.8)	13	94.6 (88.0–97.7)	88.2 (81.7–92.4)
	outliers	14	86.7 (82.9–89.8)	39.5 (0.0–67.9)	13	95.5 (93.3–97.0)	21.3 (0.0–58.7)
**E7**	overall summary estimate	17	67.0 (63.2–70.6)	37.1 (0.0–64.8)	14	88.5 (77.9–94.4)	90.4 (85.7–93.6)
	cancer-free controls	9	67.3 (62.0–72.2)	0.0 (0.0–64.8)	9	81.4 (70.3–89.0)	70.2 (40.9–85.0)
	small studies (<25)	12	67.3 (63.9–70.5)	30.9 (0.0–65.1)	10	90.8 (78.0–96.5)	91.4 (86.3–94.6)
	outliers	15	67.9 (65.2–70.5)	0.0 (0.0–51.1)	11	89.7 (82.1–94.3)	77.5 (60.0–87.4)

k = number of studies used to determine summary estimates for sensitivity and specificity.
